# The effect of practicing sports on the body composition and physical fitness of people with intellectual and developmental disabilities

**DOI:** 10.3389/fpsyg.2025.1654598

**Published:** 2025-11-26

**Authors:** Susana Diz, Miguel Jacinto, Aldo M. Costa, Rui Matos, Diogo Monteiro, José E. Teixeira, Raúl Antunes

**Affiliations:** 1 Department of Sport Sciences, University of Beira Interior, Covilhã, Portugal; 2 Research Center in Sport Sciences, Health Sciences and Human Development (CIDESD), Vila Real, Portugal; 3 Department of Sport, Exercise, and Health, School of Education and Social Sciences (ESECS), Polytechnic University of Leiria, Leiria, Portugal; 4 Department of Sports Sciences, Polytechnic Institute of Bragança, Bragança, Portugal; 5 Department of Sports Sciences, Polytechnic Institute of Guarda, Guarda, Portugal; 6 SPRINT-Sport Physical Activity and Health Research and Innovation Center, Rio Maio, Portugal

**Keywords:** intellectual disability, sedentary lifestyle, sports, physical fitness, body composition

## Abstract

**Introduction:**

People with IDD tend to have a sedentary lifestyle, which affects their health and physical fitness.

**Methods:**

The aim of this study was to analyses the effect of a sport-based intervention, with weekly 60-min sessions over 36 weeks, on the body composition and functional physical fitness of people with Intellectual and Developmental Disabilities (IDD). The sample consisted of 36 institutionalized participants with IDD, divided into two groups: experimental group with 23 participants (*M* = 37.26; SD = 13.84) and control group with 13 individuals (*M* = 38.31; SD = 14.22). A stadiometer, bioimpedance equipment and the Hand Grip, Sit and Stand, Timed Up and Go and 6 Min Walk tests were used to assess the variables of interest.

**Results:**

The experimental group showed statistically significant values in body mass index (*p* = 0.01; *r* = 0.301), muscle mass (*p* < 0.01; *r* = 0.431), Sit and Stand Test (*p* = 0.01; *r* = 0.324) and 6 Min Walk Test (*p* < 0.01; *r* = 0.399).

**Discussion:**

The results suggest that long-term programs based on the practice of sports can bring benefits to the body composition and functional physical fitness of adults with IDD.

## 1 Introduction

Intellectual and developmental disability (IDD) is defined as a developmental disorder characterized by limitations in intellectual functioning and adaptive behavior, with an impact on conceptual, practical and social domains (Schalock et al., 2021). IDD manifests itself by the age of 22 and can vary in severity from mild to profound (Schalock et al., 2021). In addition, people with IDD tend to have difficulties in executive functioning (Rodrigues et al., 2019) and motor performance, namely mobility limitations (Cleaver et al., 2009) resulting from lower tonic and muscular performance, associated with sensory deficits and slower and more imprecise motor responses (Carmeli et al., 2008), which compromise their autonomy in activities of daily living and social participation (Enkelaar et al., 2013).

At the same time, there is a high prevalence of comorbidities such as overweight/obesity, type II diabetes, hypertension and cardiovascular diseases (Calders et al., 2011; O’Leary et al., 2018), resulting from risk behaviors such as a sedentary lifestyle (Bergström et al., 2013; O’Leary et al., 2018) and reduced physical activity (PA) (Bossink et al., 2017; Pierce and Maher, 2020). These behaviors contribute to low levels of physical fitness, namely reduced levels of strength, aerobic capacity, balance and flexibility (Wouters et al., 2017) with negative consequences for walking (Cleaver et al., 2009), postural control (Lahtinen et al., 2007), manipulation of objects (Enkelaar et al., 2013) and consequently success in performing activities of daily living (Oppewal et al., 2014). This decrease in muscle strength, especially in the lower limbs, is closely associated with a decline in physical and functional capacity (Mendonca et al., 2013).

Scientific evidence has highlighted the benefits of PA and physical exercise (PE) in the prevention and control of various chronic diseases (Calders et al., 2011), as well as in improving physical fitness, namely improvements in lower limb strength, cardiorespiratory fitness (Diz et al., 2021; Jacinto et al., 2023a; Jacinto et al., 2023b; Obrusnikova et al., 2021) and body composition, decreasing fat mass and increasing muscle mass (Jacinto et al., 2023a; Obrusnikova et al., 2021). Even though research into the practice of sports in this population is limited (Diz et al., 2024b), studies carried out with other populations (e.g., elderly; visually impaired) show similar benefits (Pedersen et al., 2017).

The low adherence of people with IDD to PA and PE programs is partly a consequence of the scarcity of programs adapted to the needs and interests of this population, as well as the absence of structured progression in activities (Obrusnikova et al., 2022) and the lack of guidance from qualified professionals with experience in the field (Jacinto et al., 2021a). In addition, the shortage of motivation for regular practice is also a major obstacle (Bossink et al., 2017), highlighting the need for more participant-centered approaches. The short duration of intervention programs and their discontinuity are also identified as barriers to participation and the effectiveness of interventions (Burns et al., 2024), which can compromise participants’ motivation and involvement. Scientific evidence suggests that longer-lasting and more structured interventions tend to promote greater adherence and be more successful (Burns et al., 2024). Additionally, participation in programs that include diverse components, such as games, can encourage the frequency of practice and physical fitness (Farías-Valenzuela et al., 2022). In the same way, programs that involve social involvement (peers and teams) are often more motivating and facilitate practice (Bossink et al., 2017; Jacinto et al., 2021b) also, participants who engaged in team sports reported higher satisfaction with their lives and greater appreciation of their living conditions (Moltó and Bruna, 2017). PA and PE programs have proven to be less motivating and interesting for people with IDD (Jacinto et al., 2023a; Jacinto et al., 2024). In this regard, and considering the points discussed above, exploring the practice of sports proved to be relevant.

Sports-based intervention programs that is structured, adapted to the characteristics and interests of the participants, respecting their needs, prioritizing progression that is in line with the participants abilities and led by professionals with specialized training and experience in the field of disability. This program seeks to respond to the barriers already identified, being a long-term program that prioritizes the participants’ preferences and needs, including their opinion on the sessions, with the aim of maintaining motivation and involvement over time.

The aim of this study is to evaluate the effect of a programs e based on the practice of sports (basketball, football, volleyball, and handball) once a week for 36 weeks on the body composition and functional physical fitness of people with IDD. Thus, the authors intend to verify the following hypotheses: (i) regular practice of sports improves body composition and physical fitness in people with IDD; (ii) these improvements are greater in the EG compared with the CG.

## 2 Materials and methods

### 2.1 Study design

This is a quasi-experimental pre-post design with a comparison group. Randomization of participants was not feasible due to logistical issues related to the institutions. The EG took part in a weekly 60-min sports-based session over 36 weeks and the CG maintained their usual daily activities without taking part in the sessions organized for the EG.

The participants in the EG and CG were assessed at two different times, before the start of the intervention program (week 0) and after its conclusion (week 37).

### 2.2 Participants

This study involved people with a diagnosis of IDD who were institutionalized in two institutions in the region of Leiria, Portugal.

Eligibility criteria were defined for inclusion in the sample, namely: (1) clinical diagnosis of IDD; (2) age between 18 and 65; (3) ability to carry out the assessment procedures and (4) attendance at least 75% of the sessions. Exclusion criteria included: (1) medical contraindications for practicing sports or PE (e.g., uncontrolled cardiovascular and pulmonary diseases); (2) presence of comorbidities such as cerebral palsy, motor disability, blindness or deafness; (3) absence of functional communication skills and (4) failure to provide a duly signed informed consent form.

It should be noted that if participants in the EG did not attend at least 75% of the sessions, as stipulated in the above criteria, they would not be considered for the study, although they could continue to take part in the intervention program. It should also be mentioned that after the end of the study, the CG were given the opportunity to take part in an intervention program identical to the one applied to the EG.

The convenience sample was initially composed of 47 participants, 32 of which were in the EG and 15 in the CG. The difference in numbers between the two groups is related to the fact that most participants showed an interest in taking part in the intervention.

**FIGURE 1:**
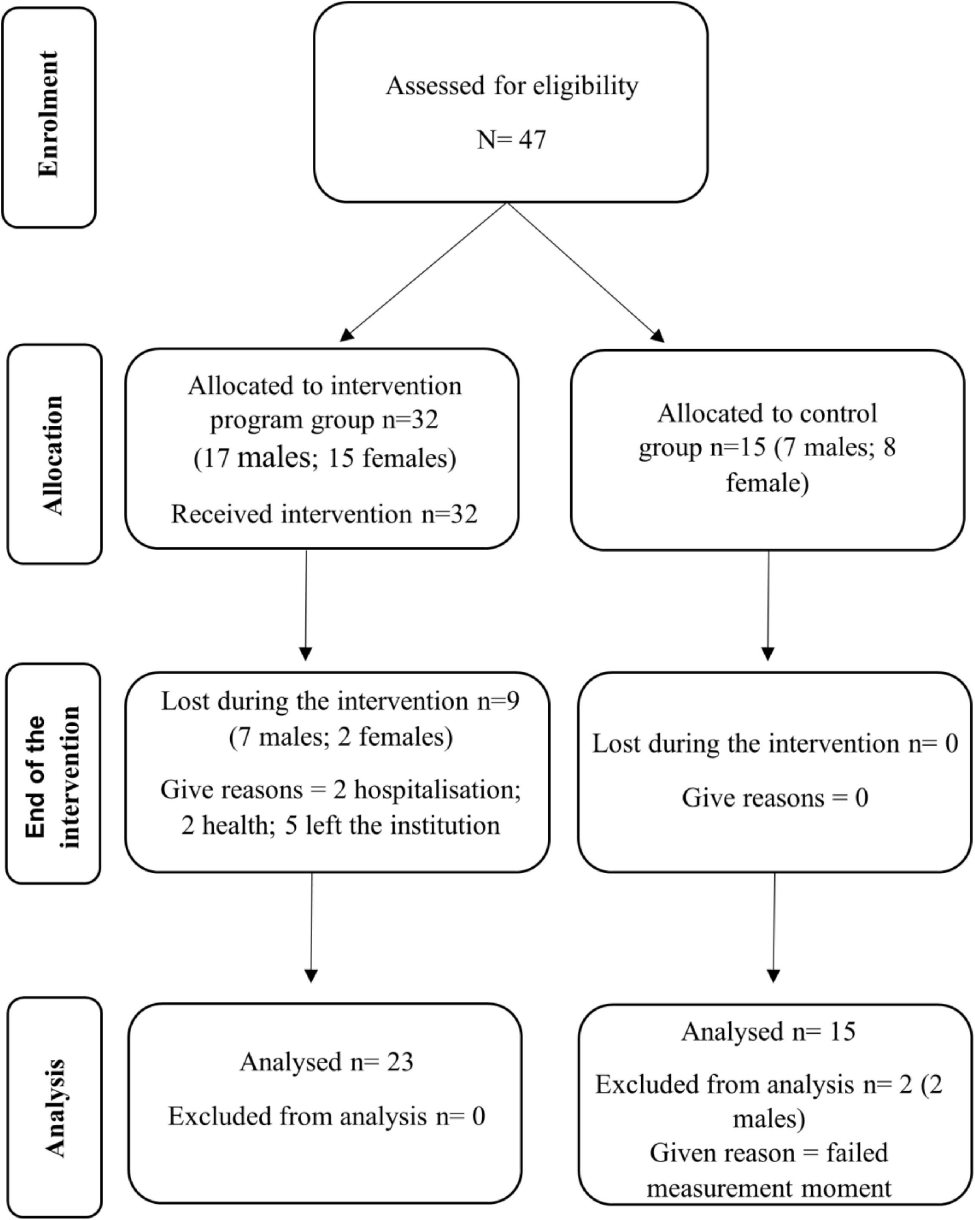
Flowchart of participants.

During the 36 weeks of the intervention, 9 members of the EG dropped out of the study for reasons unrelated to the program, two participants were hospitalized, two had health problems and the rest were no longer affiliated with their respective institutions. In the CG, two participants did not complete the final assessments and were therefore excluded from the final sample (Figure 1). Thus, the final number of participants totaled 36 individuals, of which 23 (average age: 37.26 ± 13.84) were in the EG and 13 (average age: 38.31 ± 14.22) in the CG (Table 1).

**TABLE 1:** Characteristics of participants.

Groups	*N*	Age	Height	BMI
Experimental	23	37.26 ± 13.84	159.14 ± 11.11	26.11 ± 5.27
Control	13	38.31 ± 14.22	159.27 ± 9.82	28.02 ± 5.51

The G*Power software was used, utilizing the ANOVA statistical test of repeated measures with pairwise interaction, in accordance with the literature, with the aim of calculating the number of individuals needed to detect an average effect of 0.40 (α = 0.05, 1-β = 0.95). Considering that 24 individuals (total of both groups) are needed for the effect, to predict dropouts, the authors determined a minimum number of 47 participants.

### 2.3 Instruments

#### 2.3.1 Anthropometry and body composition

A stadiometer (Seca 213) was used to measure the height of the participants. The participants were standing barefoot on the platform, leaning against the post of the stadiometer, looking forwards and with their upper limbs alongside their bodies.

A bioimpedance device (Tanita BC-50, Arlington Heights, IL, United States) was used to collect data on body composition, namely weight, body mass index (BMI), muscle mass (kg), and fat mass (kg). For proper data collection, the participants had to stand barefoot in the equipment, with their feet in contact with the electrodes.

#### 2.3.2 HandGrip

To measure upper limbs strength, the handgrip test was carried out using a hand-held dynamometer (CAMRY EH101). Its reliability and validity were confirmed by Oppewal and Hilgenkamp (2020) and Cabeza-Ruiz et al. (2019) and the procedures recommended by the Brockport Fitness Test Manual (Winnick and Short, 2014) were used.

For the test to be carried out correctly, the participants had to place their upper limbs along their body and perform the handgrip. The test was carried out for each of the upper limbs, interspersed, with 3 attempts each and the record was made by calculating the average between the three values, as recommended in the Brockport Fitness Test Manual (Winnick and Short, 2014).

#### 2.3.3 Functional capacity

For assessment of the participants’ physical fitness, the Fullerton functional test battery was applied (Rikli and Jones, 2013).

To assess the strength and endurance of the lower limbs, the “sit and stand” test was carried out, which is viable and reliable for people with IDD (Hilgenkamp et al., 2012; Wouters et al., 2017). To perform it correctly, participants had to be sitting in the center of the chair with their back straight, feet shoulder-width apart and fully supported on the floor. When the instructor gave the “start” signal, the participant had to stand up, perform the maximum extension (vertical position) and then return to the starting position. The number of repetitions performed in 30 s was counted and participants were encouraged to complete as many repetitions as possible.

To assess cardiorespiratory capacity, the “6-min walk” test was used, which is valid and reliable for the population studied (Nasuti et al., 2013). For the test to be carried out correctly, participants had to cover the maximum distance in 6 min without running, so at the “start” signal, participants were instructed to walk as quickly as possible around the distance marked with cones. The distance covered, in meters, was recorded at the end of the test. If necessary, the participants could stop and rest.

Finally, to assess physical mobility, namely speed, agility and dynamic balance, the test “Timed Up and Go” (TUG) evaluated by Cabeza-Ruiz et al. (2020) was used. Participants had to start the test sitting on a chair, with their hands on their thighs and their feet fully flat on the floor. At the “start” signal, they had to get up from the chair and walk as quickly as possible (without running) around the cone that was 2.44 m away, return to the chair and sit down. Before the start of the test, participants should be informed that the test is assessed by calculating the time it takes them to get up from the chair until they sit down again, so when the test is over, the assessor should record the time it takes the participant to complete the test.

### 2.4 Procedures

The initial phase of the study involved direct contact with the interested institutions and potential participants, where the objectives of the research were presented and any doubts regarding the intervention program were clarified. Subsequently, the individuals who met the defined inclusion and exclusion criteria were identified and approached, with full explanations, both to them and to their families or legal representatives.

Everyone was given the informed consent document, written in clear and accessible language, which described in detail the objectives of the study, the implementation phases, potential benefits and any risks. This document also emphasized that participation was voluntary and could be terminated at any time without any consequences. In addition, the informed consent guaranteed total anonymity and confidentiality of the data collected. Based on the participants’ expressions of interest, they were allocated to one of the two groups, EG or CG. Given the interest shown by the participants, the EG had a higher number of participants than the CG. Any questions raised during this process were clarified directly by the principal investigator.

The assessments took place in the morning in an inclusive sports pavilion located in the city of Leiria, properly adapted for exercise by people with disabilities. The space allowed for a functional organization that respected the privacy of the participants.

All the assessments were carried out by the same research team to ensure uniformity in the procedure. In addition, assessments took place at the same place, on the same day of the week, and at the same time as the intervention program sessions. The initial assessment took place 1 week before the start of the intervention program (week 0) and the final assessment took place the week after the end of the program (week 37). For more details on the study protocol, please refer to the study previously carried out by the same research team (Diz et al., 2024a).

### 2.5 Intervention

The intervention program was developed, implemented and monitored by three exercise technicians with proven experience and specific training in working with people with disabilities. Considering the number of participants and their specificities, the involvement of three PE technicians made it possible to ensure adequate monitoring of the participants, namely better supervision, timely feedback and adaptation of the sessions.

During the intervention program, four team sports were practiced: handball, football, basketball and volleyball. Each sport was worked on for four consecutive weeks, corresponding to four sessions, and then practiced again after 12 weeks (during which time the other sports were developed) (Table 2).

**TABLE 2:** Intervention program protocol.

Sports discipline	Technical content	Critical components	Intensities	Duration
**Week – 0 and 1**				
Playful presentation games	–	–	Light to moderate (2–6)	60 min
**Week 2; 3; 4; 5; 18, 19; 20; 21; and 34**				
Handball	-Ball handling; -Shoot; -Passing and receiving; -Dribbling; -Defensive base position; -Offensive base position; -Formal play.	-Catching and handling the ball; -Shooting in support; -Shoulder pass and bounce pass; -Two-handed reception; -Progressive dribbling -Hand and arm placement	Moderate to vigorous (4–8)	60 min
**Week 6; 7; 8; 9; 22; 23; 24; 25; and 35**				
Football	-Ball control and handling; -Passing and receiving; -Shooting on goal; -Dribbling; -Formal play.	-Ball control and handling; -Passing with the inside of the foot; -Passing and clearing; -Receiving with the inside of the foot; -Shooting with the inside of the foot; -Shooting with the instep; -Progressive dribbling; -Progressive dribbling with changes of direction	Moderate to vigorous (4–8)	60 min
**Week 10; 11; 12; 13; 26; 27; 28; 29 and 36**				
Basketball	-Ball handling; -Passing and receiving; -Passing and clearing; -Throwing; -Dribbling; -Defensive base position; -Offensive base position; -Formal play.	-Catching and handling the ball; -Chest pass and bounce pass; -Passing and clearing; -Two-handed reception; -Support throw, free throw and pass throw; - Hand and arm placement; -Progressive dribbling, -Change of direction when dribbling and protective dribbling.	Moderate to vigorous (4–8)	60 min
**Week 14; 15; 16; 17; 30; 31; 32; and 33**				
Volleyball	-Ball handling; -Passing and receiving; -Service; -Attack; -Defensive base position; -Offensive base position; -Formal play.	-Touch support pass; -Overhead pass and headline; -Service underneath; -Rematch; -Positioning on the block.	Moderate to vigorous (4–8)	60 min

As for the organization of the sessions, they were structured systematically, starting with a welcome moment that prioritized the technician-participant relationship, where everyone had the opportunity to express how they felt that day and share events from their week/weekend, followed by a brief overview of what the session would be about. This was followed by the warm-up, in which exercises directly related to the sport to be explored in that day’s session were introduced, followed by the fundamental part, centered on the practice of critical components of the sport in focus, and a final game moment, aimed at introducing the formal game of the sport. At the end of the session, in the last 5–10 min or so, the participants had some free time to experiment independently with different sports materials, promoting autonomy, exploration and active involvement.

Each session ended with a moment of reflection, in which participants were invited to express their opinion on the experience. To do this, a simple, visual method was used in which participants were asked to place a bottle cap in one of the three available boxes, each representing a different level of satisfaction (green smile for a positive evaluation, yellow for neutral and red for negative).

The instructions for the exercises were given directly, using demonstrations to make them easier to understand and carry out. Constant encouragement, as well as individualized correction tailored to each participant’s performance, were central aspects of the sessions.

The participants’ perception of physical effort was assessed after each session, specifically after the “final game,” using the Modified Borg Scale (Borg, 1998), which ranges from 0 (no effort) to 10 (maximum effort) and the Talk Test, a practical and validated tool for estimating exercise intensity in clinical and research contexts (Cowan et al., 2012; Foster et al., 2008; Persinger et al., 2004).

For more details on the intervention program, we recommend analyzing the study protocol previously published by the team (Diz et al., 2024a).

### 2.6 Statistical analysis

Regarding data analysis, we conducted a descriptive analysis of the sociodemographic data, using mean values and frequency analysis. Firstly, the normality of the distribution of the variables was assessed using the Shapiro-Wilk test (*n* < 50). Descriptive analyses were then carried out, employing measures of central tendency and dispersion, including the median and interquartile range (IQR).

As the assumptions for using parametric statistics were not met. The Wilcoxon test was used to signed-rank test to compare pre- and post-intervention values within the EG and CG (hypothesis i) and the Mann-Whitney U test was used to compare the pre-post change scores between the EG and the CG (hypothesis ii). The size of the r effect (suitable for the Wilcoxon test, allowing the comparison of two paired groups) was calculated and the reference values assumed were: “small” effect = 0.10, “medium” effect = 0.30 and “large” effect = 0.50 (Cohen, 1988; Fritz et al., 2012).

A *p*-value of *p* < 0.05 was considered to reject the null hypothesis (Ho, 2014).

The data were processed using the statistical software Statistical Package for the Social Sciences (SPSS)—V.27.0, which enabled the necessary statistical calculations for descriptive analysis, visualization, and discussion.

To test the primary hypotheses more robustly, controlling for the type I error rate, we used Bonferroni correction, which set a significance threshold of 0.0167. To be interpreted more appropriately, results with a *p*-value between 0.0167 and 0.05 should be analyzed with caution.

## 3 Results

Table 3 shows the comparison between the CG and the EG at the pre-intervention moment. As can be seen, in the initial assessment, there are no statistically significant differences between the two groups.

**TABLE 3:** Comparison between CG and EG (pre-intervention).

		CG		EG			
		Median	IQR	Median	IQR	*P*	*Z*
Body composition	BMI	23.90	10.35	28.30	7.80	0.34	-0.96
	Muscle mass	43.70	14.45	44.40	14.00	1.00	0.00
	Fat mass	12.80	17.60	22.20	19.30	0.18	-1.35
Functional physical fitness	Right HandGrip	22.00	15.10	24.00	15.70	0.56	-0.61
	Left HandGrip	19.80	8.05	20.30	14.00	0.85	-0.21
	Sit and stand test	13.00	8.00	11.00	5.00	0.23	-1.24
	TUG	6.09	4.37	5.85	3.35	0.92	-0.12
	6 min walk	491.28	166.80	468.43	191.94	0.85	-0.21

BMI, body mass index; TUG, Timed Up and Go test; 6 min walk test—distance in meters; IQR, interquartile range.

Table 4 shows the comparison between the CG and the EG at the post-intervention moment. Analyzing it, it is possible to verify that in the post-intervention period, as happened in the pre-intervention period, there were no statistically significant differences in the comparison between the two groups.

**TABLE 4:** Comparison between CG and EG (pos-intervention).

		CG		EG				
		Median	IQR	Median	IQR	*P*	*Z*	*r*
Body composition	BMI	26.40	9.15	28.60	8.90	0.20	-1.29	0.046
	Muscle mass	44.70	14.75	45.30	14.80	0.72	-0.36	0.004
	Fat mass	15.50	16.40	20.00	21.20	0.15	-1.45	0.058
Functional physical fitness	Right HandGrip	17.80	14.85	21.60	18.90	0.31	-1.02	0.029
	Left HandGrip	18.70	11.50	19.70	16.90	0.95	-0.07	0.000
	Sit and stand test	14.00	6.00	13.00	7.00	0.94	-0.08	0.000
	TUG	7.17	3.52	6.21	2.71	0.36	-0.94	0.025
	6 min walk	418.6	151.96	536.98	191.94	0.15	-1.15	0.037

BMI, body mass index; TUG, Timed Up and Go test; r, effect size; IQR, interquartile range.

The comparison between the pre-intervention (moment 0) and the post-intervention (moment 1) for the EG can be analyzed in Table 5.

**TABLE 5:** Comparison between pre and post intervention moments in the GE.

		Moment 0 (pre-intervention)		Moment 1 (post-intervention)				
		Median	IQR	Median	IQR	*P*	*Z*	*r*
Body composition	BMI	28.30	7.80	28.60	8.90	0.01*	-**2.63**	**0.301**
	Muscle mass	44.40	14.00	45.30	14.80	< 0.01*	-**3.15**	**0.431**
	Fat mass	22.20	19.30	20.00	21.20	0.38	-0.89	0.034
Functional physical fitness	Right HandGrip	24.00	15.70	21.60	18.90	0.81	-0.24	0.003
	Left HandGrip	20.30	14.00	19.70	16.90	0.38	-0.88	0.034
	Sit and stand test	11.00	5.00	13.00	7.00	0.01*	-**2.73**	**0.324**
	TUG	5.85	3.35	6.21	2.71	0.54	-0.61	0.016
	6 min walk	468.43	191.94	536.98	191.94	< 0.01*	-**3.03**	**0.399**

BMI, body mass index; TUG, Timed Up and Go test; r, effect size; IQR, interquartile range.

*p < 0.05. The bold values represents *p* < 0.05 and a statistically significant difference.

When comparing the pre-intervention moment with the post-intervention moment in the EG, it is possible to verify that it presents significant improvements in the variables muscle mass (*p* < 0.01; *r* = 0.301), sit and stand test (*p* = 0.01; *r* = 0.324) and 6 min walk (*p* < 0.01; *r* = 0.399), all with a medium effect size. In the BMI variable, a significant difference is also noticeable (*p* = 0.01; *r* = 0.301), with a medium effect size, indicating that the variable under study did not undergo a positive change.

Table 6 represents the comparison between the pre-intervention (moment 0) and the post-intervention (moment 1) for the CG.

**TABLE 6:** Comparison between pre and post intervention moments in CG.

		Moment 0 (pre-intervention)		Moment 1 (post-intervention)				
		Median	IQR	Median	IQR	*P*	*Z*	*R*
Body composition	BMI	23.90	10.35	26.40	9.15	0.60	-0.53	0.022
	Muscle mass	43.70	14.45	44.70	14.75	0.28	-1.10	0.093
	Fat mass	12.80	17.60	15.50	16.40	0.58	-0.56	0.024
Functional physical fitness	Right HandGrip	22.00	15.10	17.80	14.85	0.02*	-**2.44**	**0.458**
	Left HandGrip	19.80	8.05	18.70	11.50	0.06	-1.85	0.263
	Sit and stand test	13.00	8.00	14.00	6.00	0.66	-0.43	0.014
	TUG	6.09	4.37	7.17	3.52	0.31	-1.01	0.078
	6 min walk	491.28	166.80	418.16	151.96	0.88	-0.16	0.002

BMI, body mass index; TUG, Timed Up and Go test; r, effect size; IQR, interquartile range.

*p < 0.05. The bold values represents *p* < 0.05 and a statistically significant difference.

As can be seen in the table mentioned, when comparing the pre-intervention and post-intervention moments for the CG, statistically significant differences were observed in the handgrip test on the right hand (*p* = 0.02; *r* = 0.458), with a medium effect size, suggesting a decrease in performance.

Although the differences are not statistically significant, the comparison between the two moments reveals an increase in the median of the sit and stand test, suggesting a decrease in test performance. Similarly, it is possible to verify an increase in the median for the BMI and fat mass variables of body composition.

All GE participants evaluated the sessions, considering their level of satisfaction, and 94% of participants expressed high satisfaction with the sessions, 4% gave an intermediate evaluation and only 2% indicated that they did not enjoy the sessions.

## 4 Discussion

The main aim of this study was to analyze the effect of a 36-week sports-based program on the body composition and functional fitness of people with IDD.

Considering the results obtained, when comparing the EG and CG at the post-intervention moment, no statistically significant differences were found between the groups. However, when comparing the pre- and post-intervention moments (moment 0 and moment 1) in the EG, it was possible to see that the participants showed significant improvements in the BMI and muscle mass variables relating to body composition and in the sit and stand and 6-min walk tests relating to functional physical fitness. Regarding the CG, there was a statistically significant difference in the comparison between the two moments (moment 0 and moment 1), specifically in the handgrip test, with the right hand showing a decrease in values.

These results seem to confirm the hypotheses initially established, showing that: (i) the sports participation improves body composition and physical fitness in people with IDD and (ii) these improvements are greater in the EG compared with the CG.

With regard to the body composition of the participants in the EG, it is possible to see a significant increase in muscle mass and a significant increase in BMI when comparing the time points, however, although the value is not significant, it is also possible to see a decrease in the participants’ fat mass. BMI is calculated using the formula weight divided by height squared, and can increase due to high amounts of muscle mass and/or fat mass. Therefore, an individual with high muscle mass can have a high BMI, even if their body fat is not excessive (Rezende et al., 2010). Thus, the values obtained seem to indicate that the increase in BMI can be justified by the increase in muscle mass, considering that it has increased, it is normal that the participants’ weight has also increased and, consequently, their BMI, despite the slight decrease in fat mass values.

One possible explanation for the lack of a statistically significant difference in the fat mass variable, which is part of body composition, could be related to the fact that variables such as diet were not controlled. Considering that previous studies report that the average calorific ingestion tends to be higher in the IDD population, this is a factor that could be considered relevant (Hoey et al., 2017).

The result achieved for the muscle mass variable seems to be corroborated by the study carried out by Tomé et al. (2024), where the authors found a statistically significant improvement in the muscle mass (*p* = 0.05) of the participants. The authors applied a physical exercise intervention program, using recreational games from team sports, once a week and lasting 60–90 min, to adults with IDD. Considering the type of intervention and the sample of the study carried out by Tomé et al. (2024), the results they obtained are of particular interest.

The statistically significant difference in the sit and stand test between moment 0 and moment 1 for the EG suggests that the intervention program contributed to an increase in lower limb strength and endurance. This result can be explained by the exercises carried out during the sessions, namely exercises that involve constant movement, rapid changes of direction and lower limb thrusting, as is the case with various exercises carried out in football, basketball, handball and volleyball. The results obtained are in line with those of the study carried out by Wang et al. (2022), in which the authors implemented a physical activity intervention program for 12 weeks, twice a week, using different aerobic games for children with IDD and found significant improvements in the sit and stand test (*p* = 0.001). The same occurred in the study carried out by Calders et al. (2011), where the authors found statistically significant differences in the strength and endurance of the lower limbs, using the sit and stand test (*p* = 0.03) after a combined intervention program of strength training and aerobic training, lasting 20 weeks, twice a week, in adults with IDD. In addition, Pedersen et al. (2017), when carrying out an intervention program based on team sports with an elderly population, found statistically significant differences in the sit and stand test (*p* < 0.05), suggesting that it contributed to improving the strength and endurance of the lower limbs.

Our results in the 6-min walk test also showed a statistically significant difference when comparing the two moments in the EG, suggesting that the intervention program contributed to improving the participants’ cardiorespiratory capacity. These results are in line with those presented by Wang et al. (2022), in which the authors found statistically significant improvements in the 6-min walk test, with an increase in the distance covered by children with IDD, after the implementation of a 24-session intervention program, carried out twice a week, using aerobic games (Wang et al., 2022). Also, in the study carried out by Diz et al. (2021) with adults with DID, the participants in the EG showed a statistically significant improvement in cardiorespiratory capacity after implementing a 12-week, twice-weekly adapted physical activity program. The same occurred in the study by Obrusnikova et al. (2021), where the authors evaluated the effect of a multicomponent resistance training intervention on the strength and functional performance of adults with IDD, and found a statistically significant improvement in the 6-min walk test (*p* < 0.05), suggesting an increase in cardiorespiratory capacity. Also, in the study by Kocić et al. (2017), the authors found that the EG showed a 10% increase in the distance covered in the 6-min walk test (*p* < 0.05), after the implementation of an adapted basketball intervention program in adolescents with IDD, lasting 8 weeks and 4 times a week.

As mentioned in the previous paragraph, the difference seen in the 6-min walk test seems to indicate that the intervention program contributed to an increase in the participants’ cardiorespiratory capacity. These results may be related to the fact that the participants tended to lead sedentary lifestyles, and that regular sports practice was included in their routine, since there is a continuous stimulus for developing this capacity. In addition, team sports, such as those practiced during the sessions, involve constant movement, changes of direction and moments of running, which promote cardiovascular and respiratory adaptations (Yue and Hong, 2023). It should also be noted that, as we have already seen, there was an improvement in the strength and endurance of the lower limbs, which may have contributed to the participants being able to cover a greater number of meters more efficiently and with less fatigue in the lower limbs.

This set of abilities, related to lower limb strength and endurance, as well as cardiorespiratory capacity, tends to contribute to daily functional performance, with positive repercussions on activities of daily living, sitting down and standing up from chairs, going up and down stairs, and reducing the risk of falls (Aslan et al., 2025; Calders et al., 2011; Delgado-Lobete et al., 2021; Obrusnikova et al., 2021).

Regarding the CG, it was possible to see a statistically significant difference when comparing the two moments in the handgrip test for the right hand. Since the CG participants maintained their usual daily activities, the reason for this difference is not obvious. However, we can say that the intervention program seems to have contributed to maintaining strength in the upper limbs of the EG, as there were no significant changes in the handgrip tests, unlike the CG.

One possible explanation for the lack of significant differences in the other physical fitness variables in the EG may be related to the weekly frequency of the sessions. Although one session a week is beneficial and has brought benefits in several of the variables under study, it may not have been enough to promote improvements in upper limb strength and agility, balance and speed. Although some of the sports worked on during the sessions included the use of the upper limbs (e.g., throwing in handball or basketball), the intensity, frequency and specificity of the stimulus may not have been sufficient to bring about measurable gains in handgrip strength. Increasing the weekly frequency could lead to improvements in these variables.

Despite the obvious difficulties in executing exercises orientated toward gripping balls, dribbling and throwing, it is worth highlighting the improvements seen over the course of the sessions, where a more efficient grip was visible, as well as more precise and controlled throws. Despite the limitations, these improvements were also seen in exercises where ball handling with the feet was prioritized, as in the case of football. Even though they are not quantifiable improvements, it is pertinent to mention them, especially as they are related to exercises that require the simultaneous performance of two or more tasks and this is a difficulty experienced by this population (Jankowicz-Szymanska et al., 2012; Martin et al., 2010).

As well as analyzing the effect of sports on the body composition and physical fitness of adults with IDD, the intervention program had the additional objectives of mitigating barriers to the practice of PA, PE and sports, as well as valuing the preferences and needs of the participants, including their opinion of the sessions. In this context, the evaluation of participant satisfaction revealed extremely positive feedback on the sessions (94.10%), suggesting that the intervention contributed to the motivation and ongoing involvement of the participants. This can be supported by the absence of dropouts attributed to the program itself, contrary to evidence presented by other studies, which report the low participation of people with IDD in PA and PE intervention programs (Gjestvang et al., 2020). Monitoring participants’ satisfaction allows PE technicians to understand which exercises, activities, games and modalities participants enjoy the most, analyze the sessions based on this feedback and make the necessary adjustments. These adjustments should preserve the fundamental components to be developed but seek to align with the interests and preferences of the participants, promoting their inclusion, active listening and motivation.

The high level of adherence to the program and the results achieved reinforce the importance of integrating this type of intervention into institutional and community contexts. It is therefore essential that exercise instructors, institutions and local authorities actively contribute to its implementation and, just as importantly, to its continuity over time.

In short, the results obtained suggest that the intervention program, based on the practice of sports, contributed to an improvement in the variables of muscle mass, strength and endurance of the lower limbs and cardiorespiratory capacity.

### 4.1 Limitations and future recommendations

As with any study of a similar nature, this one also has some limitations that must be considered.

These include the periodicity of the sessions, mentioned above. Despite the benefits of holding a weekly session and the long duration of the program, for logistical reasons, namely space management and coordination with the institutions’ program of activities, it was not possible to hold a greater number of weekly sessions. Therefore, as recommendations for future studies, we suggest at least holding bi-weekly sessions.

Another relevant limitation concerns the lack of control of external variables. The fact that variables such as diet, medication and the amount of PA performed in an institutional context were not controlled are limitations to consider. It is therefore suggested that future research include control of these variables and implementing randomized designs to strengthen causal inference. In addition, unfortunately, logistical, material and financial constraints made it impossible to use more robust methods for monitoring the intensity of the intervention. For future studies, we recommend the use of more robust monitoring methods, if possible. The scarcity of studies exploring the impact of sports-based programs on the body composition and functional physical fitness of the population is notorious, so comparing the results obtained is limited. It is therefore suggested that more studies be carried out, particularly longitudinal and follow-up studies that contribute to research in this area. Finally, it is recommended that future research consider more representative samples and include different degrees of severity of IDD.

Considering the factors presented, the results of this study should be interpreted with caution, including the p-values of the BMI and TUG tests, as they are close to the value defined with the Bonferroni correction (0.0167).

## 5 Conclusion

The 36-week weekly intervention showed statistically significant improvements in body composition, particularly muscle mass, as well as lower limb strength and endurance, cardiorespiratory capacity and functional physical fitness. These results are particularly relevant considering the motor and functional difficulties often associated with this population, thus reinforcing the role of sports as an asset for promoting health, physical fitness and quality of life.

The evaluation of the participants’ satisfaction indicated that the vast majority of the sessions were enjoyed, a factor that may have contributed to their increased motivation and, consequently, to the low drop-out rate. It should be noted that none of the participants left the intervention for reasons related to the program.

The results achieved and adherence to the program support the importance of including this type of intervention in institutional and community contexts. In this sense, it is important that PE technicians, institutions and local authorities reflect on the importance of these programs, recognize their value and contribute to their development.

In short, this study aims to contribute to the advancement of knowledge in the field of sports science by demonstrating that structured sports programs, implemented on a regular basis, with a focus on the individual, can bring benefits in terms of body composition and functional physical fitness for people with IDD, paving the way for future research and the implementation of more inclusive and effective policies.

## Data Availability

The raw data supporting the conclusions of this article will be made available by the authors, without undue reservation.
